# Eczematous liraglutide eruption managed by dupilumab: A case report

**DOI:** 10.1016/j.jdcr.2024.08.018

**Published:** 2024-09-02

**Authors:** Claudia Paganini, Alfredo Belcastro, Angela Fico, Marina Talamonti, Luca Bianchi, Marco Galluzzo

**Affiliations:** Department of Systems Medicine, University of Rome Tor Vergata, Rome, Italy

**Keywords:** dermatological reaction, dupilumab, eczematous eruption, GLP-1 analogs, liraglutide

## Introduction

Glucagon-like peptide-1 (GLP-1) analogs, such as liraglutide and semaglutide, have become central in the management of type 2 diabetes mellitus and obesity. These agents work by mimicking the function of endogenous GLP-1, a hormone that increases insulin secretion, inhibits glucagon release, slows gastric emptying, and promotes satiety. Through these mechanisms, GLP-1 analogs not only improve blood sugar control but also contribute to weight loss, making them highly advantageous for patients with type 2 diabetes mellitus and obesity.^1^

In recent years, the GLP-1 analogs have gained significant popularity, particularly for their off-label use in weight loss. This trend highlights the growing interest in these drugs as a method for achieving and maintaining a healthy body weight, especially in the context of the global obesity epidemic.^2^

The use of GLP-1 agonists has surged significantly over the past 5 years, and their market size is projected to expand at an annual rate of 6.1% until 2027.^3^

Despite their clinical benefits, the use of GLP-1 analogs is not without adverse effects.^4^^,^^5^ In this case report, we describe an adverse cutaneous reaction to a GLP-1 analog, specifically an eczematous, prurigo-like eruption, which was successfully treated with dupilumab.

## Case report

A 56-year-old male patient with a longstanding history of allergic rhinitis and conjunctivitis since birth presented to our clinic with an eczematous prurigo-like eruption. He has no history of alcohol consumption or cigarette smoking. The patient is classified as having class III obesity, with a body mass index of 40.1.

Approximately 1 month prior to his visit, the patient began experiencing widespread nodular and ulcerated lesions on his abdomen, as well as on his upper and lower extremities. These lesions were accompanied by significant pruritus, which the patient rated as 8 on the Numerical Rating Scale (NRS). The itching was severe enough to disrupt his sleep, which he rated as a 6 on the NRS for sleep disturbance.

Upon further inquiry, it was noted that the patient had been prescribed liraglutide by his dietitian approximately 2 months earlier. Given the temporal relationship between the initiation of liraglutide and the development of skin lesions, liraglutide was discontinued. Comprehensive blood tests were conducted, including a complete blood count, liver and kidney function tests, autoimmunity panel, and total IgE levels. Specific tests for anti-BP180, anti-BP230, and anti-desmoglein 1-3 antibodies were also performed to rule out bullous diseases. All test results were within normal limits, with total IgE levels measuring below 100 UI/mL.

**Fig 1:**
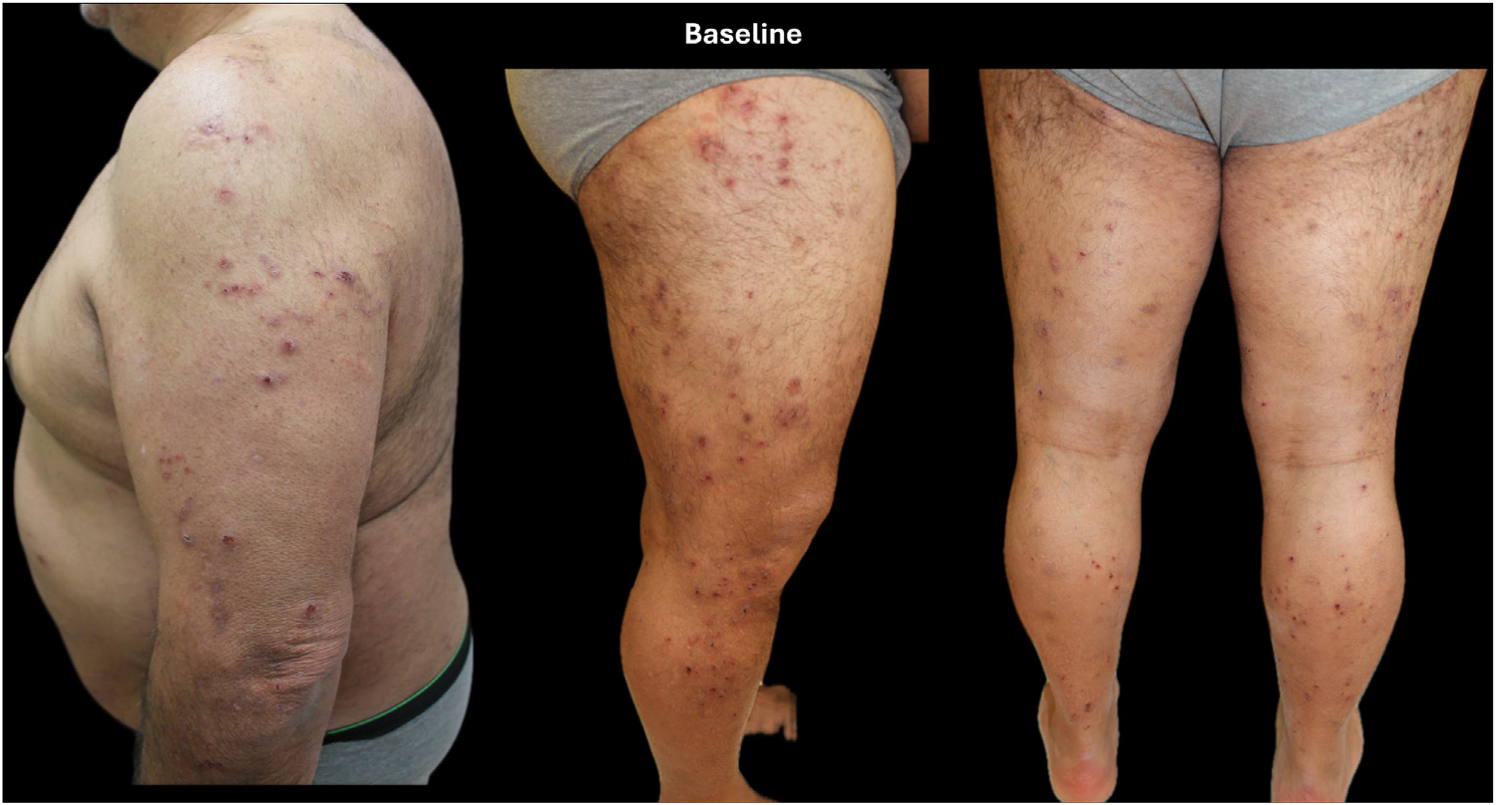
Patient at baseline.

The patient was treated with prednisone 50 mg, which was started immediately after discontinuing liraglutide and tapered weekly, along with cetirizine 10 mg twice daily for approximately 3 weeks. Despite initial clinical improvement with corticosteroid therapy, the patient continued to experience significant pruritus and eczematous lesions after tapering off the prednisone (Fig 1).

**Fig 2:**
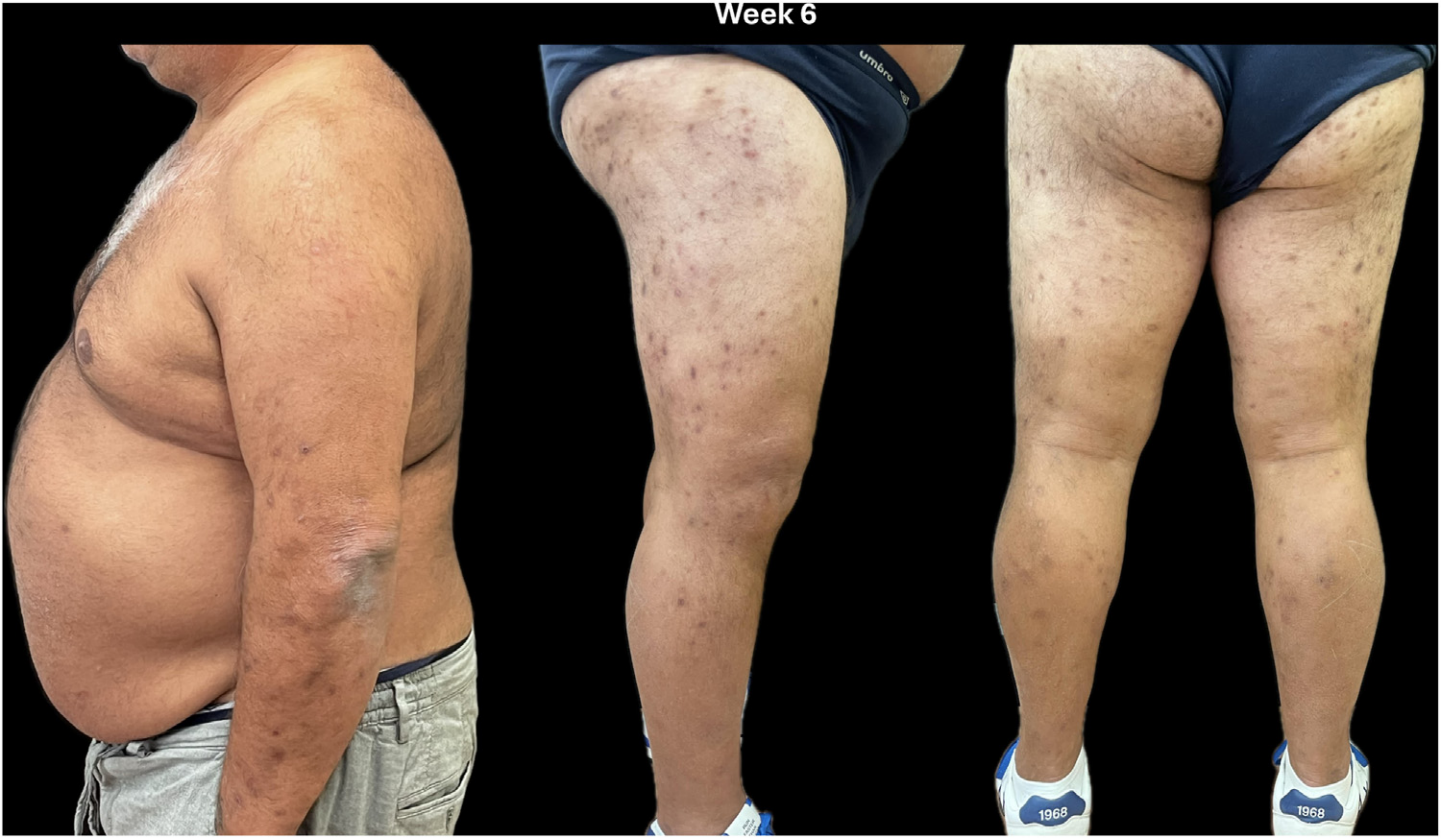
Patient at week 6 of dupilumab.

After discontinuation of systemic corticosteroids, the patient was started on the biologic medication dupilumab due to persistent severe pruritus, 3 weeks after stopping corticosteroids. An initial loading dose of 600 mg was administered, followed by a maintenance dose of 300 mg every 2 weeks. At the 6-week follow-up, the ulcerated and nodular lesions had completely resolved, leaving behind postinflammatory hyperpigmented marks (Fig 2). The patient's pruritus and sleep disturbance were both reported as 0 on the NRS. He maintained disease clearance until week 16, at which point we decided to discontinue dupilumab treatment.

## Discussion

The case report highlights a potential adverse reaction to liraglutide, an antidiabetic medication increasingly prescribed for its efficacy in managing type 2 diabetes and obesity. Despite its benefits, this case underscores the necessity for clinicians to be vigilant about potential side effects of this GLP-1 receptor agonist. While gastrointestinal issues are the most common side effects, dermatological reactions, though less frequent, can significantly impact patient quality of life.^4^^,^^5^

A review of 33 identified patients revealed several types of cutaneous reactions: dermal hypersensitivity reactions 33.3%, eosinophilic panniculitis 30.3%, bullous pemphigoid 9.1%, morbilliform drug eruptions 6.1%, and angioedema 6.1%.

Liraglutide was implicated in 6 cases, with exenatide extended-release being the most commonly associated GLP-1 agonist (57.6%). Most patients (30 of 33) recovered completely, although 3 reports did not provide outcome details. Permanent discontinuation of the offending agent was common, with 2 patients undergoing successful desensitization.^4^

In this case, the patient developed a severe pruritic rash shortly after initiating liraglutide therapy, suggesting a temporal relationship between the drug administration and the onset of symptoms. The rapid development of symptoms postadministration raises the likelihood of liraglutide being the causative agent. It appears that the reaction to the drug triggered a kind of eczematization in the patient, which was partially and transiently improved by prednisone. However, upon tapering off prednisone, the severe pruritus and some eczematous lesions reappeared. These symptoms were only resolved through the action of dupilumab, which was able to interrupt the inflammatory pathway activated by the drug-induced rash.

The treatment approach in this case involved the off-label use of dupilumab, an IL-4 receptor alpha antagonist primarily indicated for atopic dermatitis.^6^ Dupilumab has shown effectiveness in managing severe pruritus associated with various dermatological conditions due to its ability to inhibit key inflammatory pathways. For example, dupilumab has been used off-label to control generalized granuloma annulare^7^ and to treat uremic pruritus, according to the experience of Silverberg JI et al.^8^ In general, it is a valuable ally against chronic pruritus.^9^ In this scenario, dupilumab not only reduced the intense itching but also accelerated the resolution of the rash, demonstrating its potential as a therapeutic option in managing adverse cutaneous reactions to medications like liraglutide.

## Conclusion

The use of GLP-1 receptor agonists, such as liraglutide, is expanding due to their effectiveness in controlling blood sugar and aiding in weight loss. As more patients are prescribed these medications, side effects, including skin reactions, may become more common. Healthcare providers must identify and treat these side effects promptly to avoid complications and ensure patient safety. The effective use of dupilumab in this case highlights its potential as a treatment for similar adverse reactions, providing a new strategy for managing medication-induced skin issues.

## Conflicts of interest

Drs Paganini, Fico, Talamonti, Bianchi, and Galluzzo have acted as speakers and/or consultants for AbbVie, Almirall, Eli-Lilly, Janssen-Cilag, LeoPharma, Novartis, and Sanofi outside the submitted work. Dr Bianchi has served as a speaker and as a consultant for AbbVie, Novartis, Janssen-Cilag, Pfizer, UCB, and LeoPharma outside the submitted work. Dr Belcastro has no conflicts of interest to declare.
